# Cell-based approaches for the mechanistic understanding of drug-induced cholestatic liver injury

**DOI:** 10.1007/s00204-025-04016-0

**Published:** 2025-03-11

**Authors:** Enrique Timor-López, Laia Tolosa, M. Teresa Donato

**Affiliations:** 1 Experimental Hepatology Unit, Health Research Institute La Fe (IISLAFE), Torre A. Instituto Investigación Sanitaria La Fe Av Fernando Abril Martorell 106, 46026 Valencia, Spain; 2 Faculty of Medicine and Dentistry, Department of Biochemistry and Molecular Biology, University of Valencia, 46010 Valencia, Spain; 3 Biomedical Research Networking Centre on Bioengineering, Biomaterials and Nanomedicine (CIBER-BBN), Carlos III Health Institute, 46022 Valencia, Spain; 4 Biomedical Research Networking Center in Hepatic and Digestive Diseases (CIBERehd), Carlos III Health Institute, 28029 Madrid, Spain

**Keywords:** Drug-induced cholestasis, In vitro, Mechanisms, Prediction

## Abstract

Drug-induced cholestasis is one of the major mechanisms implicated in drug-induced hepatotoxicity that poses a serious problem in terms of patient morbidity and mortality, healthcare system expenses and efficacy of newly developed drugs. Impaired bile acid homeostasis due to transporter alterations, hepatocellular injury or canalicular abnormalities is the most characteristic feature of cholestasis. Given the complexity of cholestasis and the different underlying mechanisms, new models and technologies that span a variety of biological processes are needed to accurately predict drugs’ cholestatic potential. This review outlines the main triggering mechanisms of drug-induced cholestasis and summarizes the currently available in vitro systems and techniques that attempt to forecast and provide mechanistic details of cholestasis caused by drugs.

## Introduction

Drug-induced liver injury (DILI) is a complex adverse reaction to prescribed drugs and other substances that poses a significant clinical issue in terms of patient morbidity and death, healthcare system costs and drug development (Fernandez-Checa et al. 2021).

The variability of the clinical symptoms, severity, and consequences of DILI is significant because a vast array of clinical manifestations is indicative of toxic liver damage, ranging from asymptomatic and temporary increases in serum levels of liver enzymes to acute liver failure. From a clinical point of view, hepatotoxicity patterns are classified as hepatocellular, cholestatic, or mixed, using consensus criteria based on relative ratios of alanine aminotransferase and alkaline phosphatase activity levels (Aithal et al. 2011; European Association for the Study of the Liver. Electronic address et al. 2019). According to Oorts et al. (2015), cholestatic injury actually accounts for up to 50% of reported DILI cases (Oorts et al. 2015). About 73% of all cholestatic DILI cases are caused by single prescription drugs, primarily antibiotics, antifungals, immunomodulators, antipsychotics, hypoglycemic drugs and steroids (Gijbels et al. 2019).

The hallmark of cholestasis is the disruption of bile flow and secretion, which results in the accumulation of bile acids (BAs) and their conjugate bile salts in the liver and/or throughout the body, and in subsequent toxicity to liver cells. Although three different types of triggering factors (changes in transporters, alterations in hepatocellular structure and changes in bile canalicular dynamics) can cause drug-induced cholestasis (DIC) (Gijbels et al. 2019), the functional alteration of hepatobiliary transport systems is considered a major mechanism. Hepatocyte transporters are essential for the movement of both BAs and drugs (Gijbels et al. 2019). Transporters situated at the basolateral membrane are vital for the absorption of chemicals from sinusoidal blood (Pauli-Magnus and Meier 2006), while canalicular transporters are responsible for the clearance and secretion of drugs, BAs and other bile components across the canalicular membrane of hepatocytes into bile (Pauli-Magnus and Meier 2006). Thus, any effect of drugs on these transporter systems can consequently result in the accumulation of potentially harmful BAs or increased xenobiotics uptake by the liver, which may subsequently lead to liver cell injury (Pauli-Magnus and Meier 2006). In fact, the ability of a compound to disrupt the bile salt export pump (BSEP) seems to be associated with its potential to cause liver injury in humans (Morgan et al. 2010). However, it is clear that DIC involves the interaction of various underlying mechanisms, and that numerous liability factors have to be taken into account when assessing a specific compound’s cholestatic potential.

Animal studies are unavoidable in both preclinical and clinical drug research, but they have significant limitations due to the considerable differences in drug metabolism and pharmacokinetics between experimental animals and humans (O’Brien et al. 2004). Thus, in past years, considerable efforts have been made to develop new in vitro systems for predicting DILI, including DIC. Certain systems, such as membrane vesicles derived from transporter-transfected cells, are particularly effective for examining the interaction between a potentially cholestatic compound and a specific transporter (Morgan et al. 2010). However, they simplify the mechanistic intricacies of DIC to the interaction of a single transporter. In contrast, other models aim to provide a more comprehensive approach because they incorporate different biological processes using distinct methods. This review presents an evaluation of the key molecular mechanisms linked with DIC, along with discussion about the various liver in vitro models and techniques presently employed to assess drugs’ cholestatic potential.

## BA synthesis and transport

**Fig. 1:**
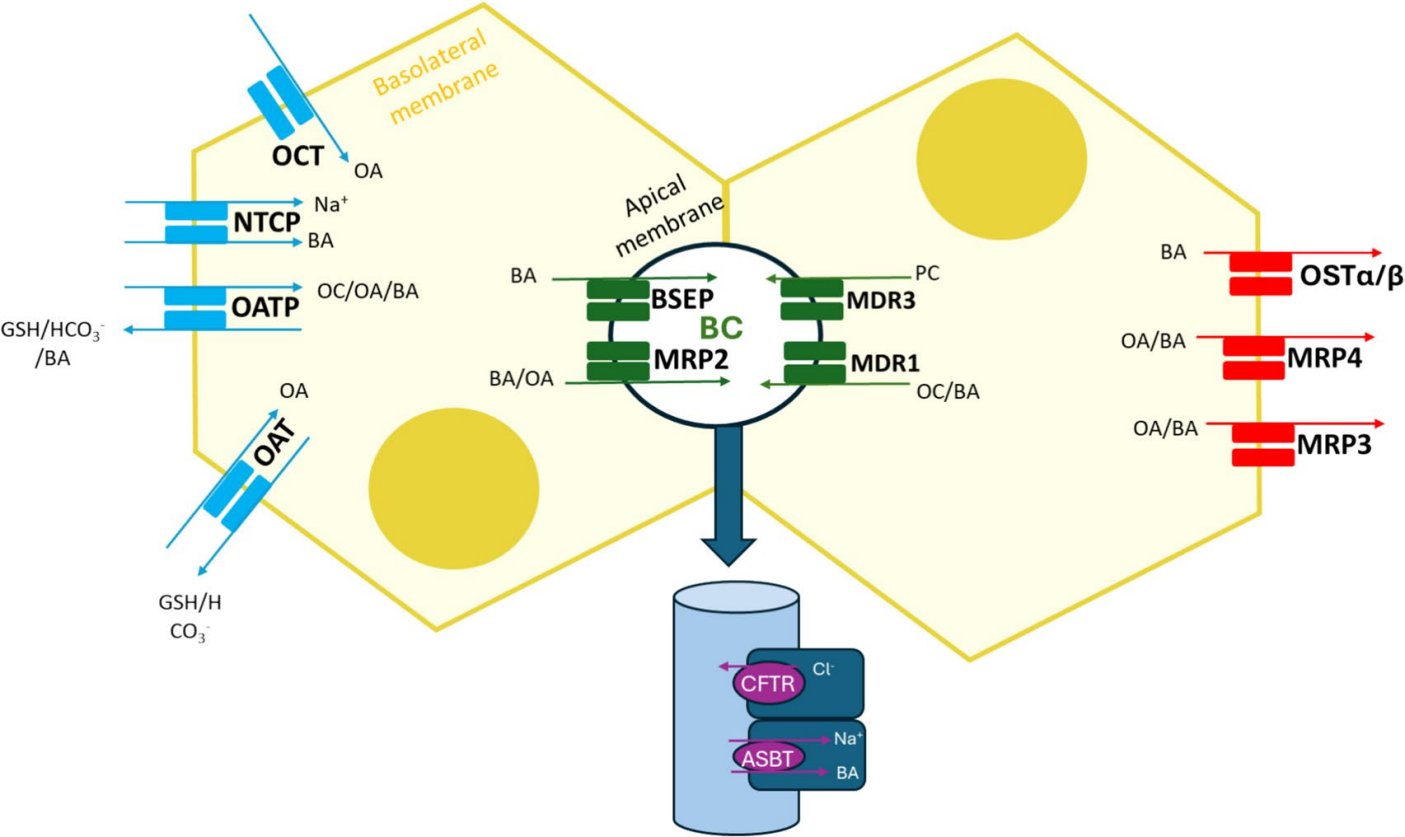
Major hepatic transporters involved in BA homeostasis. The basolateral transporters that face the hepatic sinusoid are in charge of the uptake (NTCP, OCT, OAT and OATPs) or the efflux (MRP3 and MRP4), respectively, of BAs and other molecules (organic cations and anions) from or to the bloodstream. The apical transporters that face the bile canaliculi (BSEP, MRP2, MDR1 and MDR3 are responsible for the canalicular efflux of organic cations/BAs, phospholipids and cholesterol. Cholangiocytes express the cystic fibrosis transmembrane regulator (CFTR), which is a chloride channel, and an apical Na^+^-dependent BA transporter (ASBT). *BA* bile acid, *BC* bile canaliculi, *BSEP* bile salt export pump, *GSH* glutathione, *MRP* multidrug resistance-associated protein, *NTCP* Na^+^-taurocholate cotransporting polypeptide, *OATP* organic anion transporting polypeptide, *OA* organic anions, *OC* organic cations, *OST* organic solute transporter, *PC* phosphatidylcholine

BAs, which are the principal organic solutes in bile, are de novo synthesized from cholesterol by two main pathways: the "neutral" one, which uses cholesterol 7α-hydroxylase (CYP7A1) to convert cholesterol into 7α-hydroxycholesterol; the "acidic" pathway, which is started by 27-hydroxycholesterol formation (Deferm et al. 2019a). The last BA synthesis stage entails BAs' conjugation with taurine or glycine (Fuchs 2003). Both conjugated and unconjugated BAs may go through additional sulfation or glucuronidation within hepatocytes before being released into bile canaliculi by canalicular transporters (Pauli-Magnus and Meier 2006). Figure 1 summarizes the principal transporters implicated in BA homeostasis.

BSEP and the multidrug resistance protein 3 (MDR3) are essential transporters located in the canalicular membrane that facilitate BA secretion and help to maintain low levels of harmful BAs within cells (de Lima Toccafondo Vieira and Tagliati 2014; Gijbels et al. 2019). These proteins belong to the superfamily of ATP-binding cassette (ABC) transporters, which also includes multidrug resistance-associated protein 2 (MRP2) that is responsible for the canalicular export of conjugated BAs and organic anions. Although MRP3 and MRP4, located at the basolateral membrane, scarcely contribute to BAs export under physiological conditions, they play an important role in the efflux of BA and diverse endogenous compounds and xenobiotics to the bloodstream when biliary excretion diminishes (Deferm et al. 2019a; Denk et al. 2004).

The apical sodium-dependent bile salt transporter (ASBT) and the organic anion transporting polypeptide (OATP) mediate the partial reabsorption of BAs into cholangiocytes after they are secreted (Hagenbuch and Dawson 2004). The organic solute transporter (OSTa-b/SLC51A and B) then secretes reabsorbed BAs into the peribiliary plexus, where they can be released to bile again (Ballatori et al. 2009).

The majority of primary BAs are transported to the intestine and gallbladder, where intestinal bacteria dehydroxylate them to create secondary BAs, such as lithocholic acid (LCA) and deoxycholic acid (DCA) (Deferm et al. 2019b). The BA pool is maintained in the distal intestine by reabsorbing around 90% of conjugated and unconjugated BAs by enterohepatic circulation and passive diffusion (Boyer 2013; Deferm et al. 2019b). Then, basolateral transporters promote BA reuptake from sinusoidal circulation into hepatocytes (Trauner and Boyer 2003). The Na^+^ taurocholate cotransporting polypeptide (NTCP) facilitates the transport of bile salts to hepatocytes through a Na^+^-dependent mechanism (Meier and Stieger 2002). Additionally, the sinusoidal transport of bile salts and organic anions also occurs independently of Na^+^ and is mediated by OATPs, which are high-affinity transporters that specifically mediate the uptake of unconjugated species (Hagenbuch and Meier 2003).

## Mechanisms of drug-induced cholestasis

**Fig. 2:**
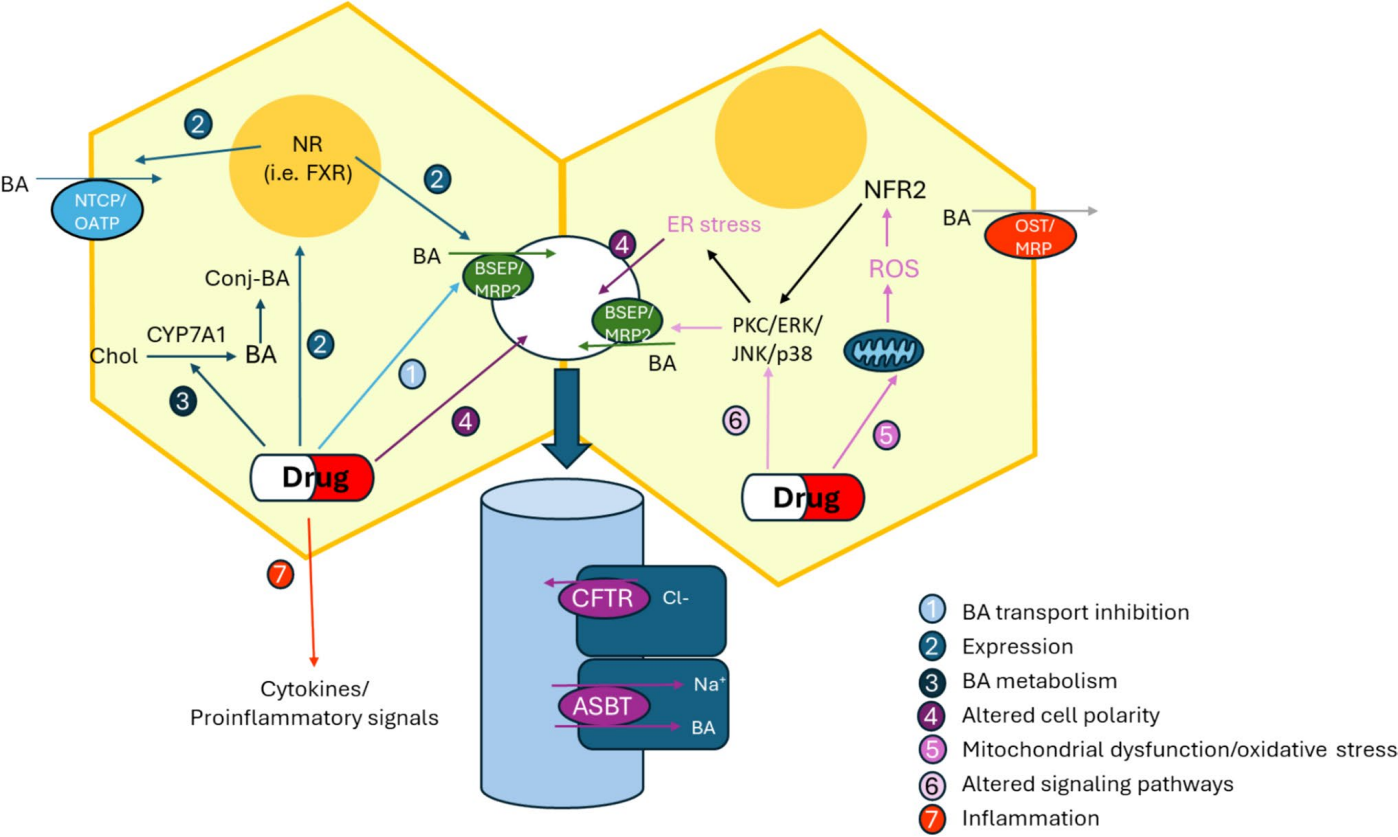
Overview of the mechanisms involved in drug-induced cholestasis. Drugs or their metabolites can impair the hepatobiliary transport of BAs by different mechanisms, including direct inhibition of the function of BA transporters (1), interaction with nuclear receptors (i.e. FXR) and alteration of the gene expression of BA transporters (2), alteration of BA metabolism by impairment of the key enzymes involved in BA synthesis and conjugation (3), disturbance of cell polarity and bile canaliculi dynamics (4), induction of mitochondrial dysfunction and oxidative stress leading to NRF2 activation and impaired expression of key BA transporters (5), activation of signaling pathways and endoplasmic reticulum (ER) stress that can impair the dynamics and dilatation of bile canaliculi or trigger apoptotic cell injury (6), and induction of the release of diverse cytokine and pro-inflammatory signals that results in inflammatory infiltration and liver damage (7). *ASBT* apical Na^+^-dependent BA transporter, *BA* bile acid, *BSEP* bile salt export pump, *CFT* cystic fibrosis transmembrane regulator, *Chol* cholesterol, *CYP7A1* cholesterol 7α-hydroxylase, *ER* endoplasmic reticulum, *MRP *multidrug resistance-associated protein, *NR* nuclear receptor, *NRF2* nuclear factor erythroid 2-related factor 2, *NTCP* Na^+^-taurocholate co-transporting polypeptide, *OATP* organic solute transporter, *ROS* reactive oxygen species

DIC can develop via different pathophysiological mechanisms. The main initiating events include transporter, hepatocellular and bile canalicular alterations. Indeed, cholestatic drugs can exert their noxious effects through many of these events at the same time. These mechanisms are closely related to key events like inflammation, mitochondrial impairment, oxidative stress, endoplasmic reticulum (ER) stress, and the adaptative response to these detrimental situations. However, whether initiating and key events are the cause or consequence among themselves is not always clear because the molecular mechanisms underlying DIC are usually interconnected in a complex manner. The list of drugs with a well-known cholestatic potential is long, and each drug exhibits its own mechanisms to generate DIC. These different ways of action make the task of deciphering the molecular basis of DIC even more challenging. Figure 2 exemplifies the major mechanisms implicated in DIC.

### Alterations of bile acid homeostasis, transport and metabolism

Drug-induced alterations of the transporters located at the basolateral or apical hepatocyte membranes can contribute to deficient BA transport (Jazaeri et al. 2021). Cholestatic drugs can inhibit BA transport through three different mechanisms: by directly operating on transporters as competitive or non-competitive ligands (ritonavir, pioglitazone, troglitazone, etc.), by altering their genetic expression (isoniazid, bosentan, lopinavir, glimepiride, etc.) or by inducing their internalization and/or degradation (rifampicin, cyclosporine, estradiol-17β-D-glucuronide, etc.) (Kralj et al. 2021).

#### Inhibition of bile acid transport

The BSEP is the main canalicular transporter responsible for exporting BAs, including unconjugated and taurine- or glycine-conjugated BAs, and is considered the rate-limiting step of bile salt secretion flow (Stieger and Geier 2011). BSEP function inhibition by drugs has been associated with their ability to induce liver injury (Morgan et al. 2013). However, some studies have shown that the in vitro inhibition of BSEP by itself is not a good predictive marker of DILI (Morgan et al. 2013; Chan and Benet 2018), which suggests that functional alterations of other BA transporters may be involved. Inhibition of other efflux transporters, such as MRP2 that is located at the canalicular membrane, or basolateral MRP3 and MRP4, may also make a contribution in DIC.

Direct inhibition of BAs transporters by drugs can be classified in *cis* or *trans*. In *cis*-inhibition, the cholestatic drug enters the hepatocyte and exerts its effect from inside the cell (rifampicin, cyclosporine, bosentan, etc.). In *trans-*inhibition, the drug needs to be first transported to bile canaliculi by a different transporter before exerting its effect on the target through its luminal side. An example of *trans*-inhibition is the effect on the BSEP of progesterone or estradiol-17β-D-glucuronide, compounds that need to be first transported to be bile canaliculi through MRP2 (Stieger et al. 2000). Moreover, some compounds can carry out both types of inhibition, which is the case of bosentan that can *cis*-inhibit BSEP and *trans*-inhibit MRP2 (Stieger and Geier 2011). It is important to note that each compound can inhibit several transporters. This may, in turn, generate the accumulation of diverse profiles of BAs with different toxic potentials. In general, the more hydrophobic (lipophilic) BA is, the more toxic its abnormal accumulation is (Schadt et al. 2016). Accordingly, LCA, DCA and chenodeoxycholic acid (CDCA) are more toxic when accumulated than ursodeoxycholic acid or cholic acid.

#### Alteration of transporter expression

Some drugs are capable of reducing the expression of the genes that codify for different transporters through the interaction with diverse nuclear receptors and associated factors, like farnesoid-X receptor (FXR). FXR is a steroid nuclear receptor that can be strongly activated by BAs and is responsible for the regulation of multiple genes related to BA metabolism. FXR can bind SIRT1 by forming the FXR/SIRT1 heterodimer, which can positively regulate the expression of BSEP (*ABCB11*) and MRP2 (*ABCC2*). Isoniazid can inhibit SIRT1 deacetylation to, thus, prevent its interaction with FXR, lead to decreased BSEP and MRP2 expression (Qu et al. 2018; Zhang et al. 2020b) and, finally, to accumulation of BAs within hepatocytes. A similar mechanism has been described for rifampicin (Wen et al. 2022). It is also known that low physiological MRP4 (*ABCC4*) expression levels (Deeley et al. 2006) markedly increase during cholestasis as a part of an adaptative response that consists in the basolateral efflux of BAs to compensate BSEP and MRP2 dysfunctions (Jetter and Kullak-Ublick 2020). In chlorpromazine-derived cholestasis, increased MRP4 expression has been observed, along with a decreased expression of NTCP (*SLC10A1*), to avoid the influx of more BAs (Antherieu et al. 2013).

There are also reports that some polymorphic forms of transport proteins can increase individual susceptibility to DIC. This is the case of the common *ABCB11* gene variant 1331 T > C, which has been associated with significantly reduced hepatic BSEP mRNA levels and an increased risk of DIC (Stieger and Geier 2011).

#### Alteration of bile acid metabolism

Not only is BA transport crucial for BA homeostasis, but also its hepatic metabolism because an imbalance in BA synthesis, conjugation and/or degradation ratios can result in toxic accumulation in the hepatocyte. Multiple cholestatic drugs participate in such dysregulation through mechanisms involving alterations to various signaling and regulatory pathways.

Once more, the previously mentioned FXR is a good example. When CDCA and other BAs bind FXR, it forms a heterodimer with small heterodimer partner (SHP) and translocates into the nucleus. The main function of an activated FXR/SHP heterodimer is the suppression of *CYP7A1*, a rate-limiting enzyme in BA synthesis (Sanoh et al. 2019). Therefore, the FXR/SHP pathway allows negative feedback regulation when BA concentration is high within hepatocytes. It has been suggested that, in addition to other cholestatic mechanisms, rifampicin is able to inhibit FXR/SHP that, in turn increases CYP7A1 enzyme activity and, consequently, synthesis and accumulation of BAs (Zhuang et al. 2022).

An in vitro study with HepaRG cells has demonstrated that potent cholestatic drugs cause intracellular accumulation of BAs by not only impairing BA transport, but by also altering BA metabolism. In particular, cyclosporine, chlorpromazine and troglitazone preferentially lead to the accumulation of unconjugated hydrophobic BAs (CDCA, DCA, LCA) by inhibiting amidation and sulfation activities (Sharanek et al. 2017). Interestingly, these hydrophobic and toxic BAs are considered relevant in the progression of BA-induced liver injury (Schadt et al. 2016).

#### Disturbance in cell polarity and bile canaliculi dynamics

Cholestatic drugs can exert their detrimental effects by altering cell polarity; that is, by inducing changes in the properties of their basolateral and apical membranes. For example, rifampicin can decrease the MRP2 function at the apical membrane by promoting both its internalization and degradation. First, this drug can activate the PKC/ERK/JNK/p38 signaling pathway that, in turn, stimulates clathrin and the AP2 complex to induce MRP2 endocytosis (Xu et al. 2020). Second, rifampicin can also increase the activity of GP78, an E3 ubiquitin ligase responsible for the ubiquitination and further degradation of MRP2 (Chen et al. 2022). These two molecular mechanisms allow rifampicin to reduce MRP2 expression at the apical membrane of the hepatocytes to, thus, decrease its capacity of evacuating BAs into bile canaliculi.

Another way to generate cholestatic damage is to impede the natural bile flow through bile canaliculi, which is necessary to allow the correct elimination of BAs. Obstruction, shrinking, loss of contractibility, or even the total disruption, of bile canaliculi can lead to accumulation of BAs. These situations can be generated by cytoskeletal changes, alterations in cell–cell junctions or accumulation of some molecule adducts in the canalicular lumen. Bile canaliculi obstruction by isoniazid has been thoroughly studied. This drug can generate different metabolites that interfere with heme synthesis, and lead to the toxic accumulation of protoporphyrin IX in bile, which results in bile canaliculi obstruction (Sachar et al. 2016).

In chlorpromazine-induced (Wu et al. 2022) and cyclosporine-induced (Sharanek et al. 2014) cholestasis, it has been observed that oxidative stress can lead to the disruption of cytoskeletal protein F-actin that, in turn, reduces peripheral bile canaliculi contractibility. It has been also proposed that pericanallicular myosin can play a role in bile canaliculi dynamic movements (Sharanek et al. 2016). This hypothesis is supported by evidence of treatment with bosentan can decrease myosin light chain kinase 2 phosphorylation that, in turn, derives in bile canaliculi dilatation (Koga et al. 2023). All these abnormal situations can compromise the normal evacuation of BAs by promoting their toxic accumulation within hepatocytes.

### Other mechanisms related to cholestasis

Besides alterations regarding transporters, BA metabolism or bile canaliculus structure, DIC is also associated with multiple mechanisms of hepatocyte damage, such as mitochondrial impairment, oxidative stress, ER stress and inflammation. Because of their essential function in cellular energy production, mitochondria are one of the most exposed and sensitive organelles to all kinds of toxic damage. Mitochondrial dysfunction plays a key role in excessive reactive oxygen species (ROS) generation and the induction of apoptotic events by toxicants, and many cholestatic drugs have been reported to produce mitochondrial damage (Zhuang et al. 2022). For instance, isoniazid can generate mitochondrial injury in four different ways: forming adducts with NAD^+^ to, thus, affect the tricarboxylic acid cycle, respiratory chain and ATP production (Lee et al. 2013; Zhu et al. 2020); affecting the proton equilibrium by mitochondrion permeability transition pore formation that, in turn, promotes apoptosis (Lee et al. 2020; Mu et al. 2020); inhibiting SIRT1 to, thus, alter mitochondrial biogenesis (Zhang et al. 2017); decreasing mitochondrial mechanisms of repair, division and fusion (Li et al. 2019).

A rise in ROS levels is one of the commonest key events to occur during DIC because diverse cholestatic drugs can generate an oxidative stress scenario. Increased ROS can cause the oxidation of proteins, lipids and nucleic acids by generating irreversible damage in multiple organelles that may, in turn, lead to apoptosis. Apoptosis can also be directly induced by cholestatic drugs. For example, in chlorpromazine-mediated cholestasis, it has been reported that accumulation of BAs can induce *DR5* gene expression and lead to the activation of the death receptor signaling pathway (Wu et al. 2022). It has been postulated that some drugs generate DIC by favoring a previous oxidative stress scenario that leads to accumulation of BAs. For instance, chlorpromazine (Antherieu et al. 2013) and clavulanate (Petrov et al. 2021) can promote rising ROS levels to, thus, trigger an adaptative response driven by nuclear factor erythroid 2-related factor 2 (NRF2). NRF2/ARE pathway activation lowers the expression of BSEP and MDR3 that, consequently, causes BA accumulation in hepatocytes, among other effects. Similarly, cyclosporine can generate an oxidative stress situation by triggering PKC/ERK/JNK/p38 signaling pathway activation and the consequent internalization of MRP2 and BSEP (Sharanek et al. 2014).

Inflammation is frequently associated with DIC, but it remains unclear whether it is a cause or consequence of cholestasis. It has been proposed that high BA levels can directly induce either chemokine production by hepatocytes (Allen et al. 2011) or a neutrophil-mediated inflammatory response that increases oxidative stress and destroys hepatocytes (Gujral et al. 2004; Jaeschke 2006). The suggested mechanism means that BAs interact with a receptor, probably TGR5 or S1PR2, to, thus, activate the JNK and ERK signaling pathways, which completely activate EGR1 and trigger the expression of multiple pro-inflammatory genes like *ICAM1*, *CXCL1*, *CXCL2* and *IL17* (Woolbright and Jaeschke 2019). Moreover, ROS overproduction can interact with the NFκB signaling pathway and mediate the expression of pro-inflammatory proteins like TNFα that, in turn, leads to an inflammation scenario in the liver, with inflammatory infiltration and damage (Zhuang et al. 2022).

Finally, ER stress is another frequently associated event with cholestasis. When ER stress begins, the unfolded protein response pathway is triggered through three different signaling pathways, namely pathways IRE1, PERK or ATF6 (Kettel and Karagoz 2024). Rifampicin has been described to induce ER stress via two different mechanisms: directly activating PXR and inducing the expression of multiple CYP genes (Szczesna-Skorupa et al. 2004), which can promote ER stress due to the activation of the IRE1 and PERK signaling pathways (Hou et al. 2022) or by also inducing cholestasis by BSEP down-regulation (Stieger et al. 2000; Wen et al. 2022).

## Cell models for the prediction of cholestasis

Much research effort has been made to develop and optimize in vitro systems to be applied to study DIC. A variety of in vitro models has been proposed, including subcellular systems, cell-based 2D models and 3D culture models with different degrees of complexity. Although each model presents singular features and inherent advantages and limitations in terms of availability, feasibility, complexity and potential applications (Table 1), they all contribute to anticipate drugs’ cholestatic potential and any underlying mechanisms.

**Table 1:** Overview of the major advantages and disadvantages of several in vitro systems proposed for the evaluation of drug-induced cholestasis

	Advantages	Limitations
Membrane vesicles (from liver tissue membranes or from transfected cell lines)	- Relatively fast and reproducible assays - Amenable for medium- to high-throughput screenings - Direct quantification of transport inhibition by drugs - Expression of multiple transporters in a native membrane environment (vesicles from liver membranes) - Commercially available (membranes from transfect cell lines)	- Vesicle preparation technically challenging and limited availability (from liver membranes) - Lack of expression of several transporters (vesicles from transfected cell lines) - Scarcity of appropriate specific probe substrates - No biotransformation capacity (no detection of effects due to drug metabolites) - Little physiological relevance
Transfected cell lines (MDCK, LLC-PK1, HEK293)	- High and stable expression of transport proteins - Allow the quantification of intracellular accumulation of probe substrates - Expression of transporters in a cell membrane environment - Amenable for uptake and/or efflux transport assays - Identification of drug effects on specific transporters - Transcellular vectorial transport studies (cell lines co-transfected with uptake and efflux transporters) - Higher sensitivity for apical transport evaluations than membrane vesicles	- Generation of transfected cell lines is technically demanding (particularly for the co-expression of more than one functional protein) - Limited number of transport proteins expressed (usually one or two) - Difficulty to estimate direct efflux (BSEP) inhibition (highly depends on uptake capacity) - Transfected cell lines are not of hepatic origin - No biotransformation capacity (effects due to drug metabolites are not detected) - Little to moderate physiological relevance
Hepatocytes in suspension	- High availability (commercially available), easy handling, and relatively low cost - High expression of basolateral uptake transporters - Possibility of using hepatocytes from different donors (individual or pooled batches) - Amenable for medium- to high-throughput assays	- Very short-term studies (viable in suspension for a few hours) - Cryopreservation may alter the function of uptake transporters - Not useful for efflux studies due to internalization of canalicular transporters
Sandwich-cultured hepatocytes	- Polarized expression of multiple basolateral and apical transporters in a native membrane environment - Functional bile canaliculi structures - Allow uptake and efflux assays, quantification of intracellular substrate accumulation and calculation of the Biliary Excretion Index of drugs - Analysis of drug effects on transport capacity at both functional and gene expression levels - Preservation of cellular regulatory pathways and the possibility of mechanistic studies - Biotransformation capacity (estimation of effects produced by metabolites) - Compatible with complementary drug toxicity studies - Relatively high physiological relevance	- Time-consuming (at least 4–6 days are needed for the optimal expression of canalicular transporters) - Relatively high cost and low availability (human hepatocytes) - Relatively low expression of basolateral uptake transporters (compared to hepatocytes in suspension) - Progressive down-regulation of drug-metabolizing enzymes (loss of biotransformation capacity) - Reproducibility of results is compromised by batch-to-batch variability and technical expertise requirements - Low- to medium-throughput screenings - Interpretation of drug effects on biliary efflux very much depends on the rate of substrate uptake by cells and its retention in bile canalicular structures
Hepatoma cell lines (HepaRG, HuH7, HepG2)	- High availability, easy handling, and relatively low cost - Stable expression of transporters in a native membrane environment and high reproducibility of results - Potential application to mechanistic studies of cholestatic drugs (HepaRG) - High expression of some drug-metabolizing enzymes (i.e. CYP3A4 in HepaRG) allows the effects of drug metabolites to be evaluated - Suitable for long-term studies (HepaRG)	- Cells of tumoral origin - Low canalicular BSEP expression and alterations in the relative expression of uptake/efflux transporters compared to hepatocytes and liver tissue - Poor biotransformation capacity (HepG2, HuH7) or altered expression of certain CYP enzymes (HepaRG) may make the interpretation of results difficult - Time-consuming differentiation procedure (HepaRG) - Low- to medium-throughput screenings - Lower physiological relevance than sandwich-cultured hepatocytes
Stem cell-derived hepatocytes	- High availability of stem cells from commercial sources - Suitable for functional transport assays and gene expression analyses - Potential application to investigate the mechanisms involved in cholestasis - Possibility of personalized studies (patient-derived cell models)	- Differentiation protocols are time-consuming and expensive, and require skilled personal - Need for optimized and standardized protocols to increase reproducibility - Immature hepatic phenotype (low expression of some drug-metabolizing enzymes and transport proteins) - Scarce information on their potential application to assess drugs’ cholestatic potential
3D culture systems (hepatocytes or hepatoma cell lines)	- Restoration of cell polarity and expression of multiple basolateral and apical transporters - Promotion of cell–cell and cell-extracellular matrix interactions - Biotransformation capacity (estimation of the effects produced by metabolites) - Preservation of cellular regulatory pathways and possibility of mechanistic studies - Applicable for long-term studies - Possibility of co-culturing with other cell types - Physiological relevance	- Preparation of some types of 3D cultures is time-consuming, has a high cost and is technically demanding - Reproducibility very much depends on multiple factors (type of 3D culture, scaffold composition, spheroid size, microfluidic device, etc. - Potential interference of biomaterials with assay tests (scaffold-based systems) - Interpreting the results from uptake and efflux assays may be complex - No easy identification of inhibitory drug effects
Liver slices	- Polarized and physiological expression of transporters - Preservation of native tissue architecture, multiple cell types, extracellular matrix and bile canaliculi structures - Suitable for biochemical assays and histopathological techniques - Retention of key signaling pathways with the possibility of mechanistic insights and the study of the involvement of each liver cell type in drug-induced cholestasis - Expression of drug-metabolizing enzymes - High physiological relevance	- Use is limited by technical, economic and reproducibility issues - Scarce availability of fresh (human) liver tissue - Only compatible with short-term studies (i.e. 24 h) - Lack of standardized liver slice culture protocols requires the extensive characterization of each preparation and impairs reproducibility - Low-throughput screenings

### Subcellular models

The subcellular systems used in preclinical transport assays include *Xenopus laevis* oocytes (Gerloff et al. 1998), inverted membrane vesicles (inside-out) from insects or mammalian cell lines overexpressing specific transporters (e.g. BSEP) (Kock et al. 2014; Morgan et al. 2010) and canalicular membrane vesicles (Horikawa et al. 2003). By means of these in vitro models, inhibition of the BSEP and other efflux transporters can be easily examined with relatively cost-effective and high-throughput assays. Inverted vesicles efflux transporters, oriented to the outward membrane surface, are readily accessible to probe substrates and test compounds. Transporter function and direct inhibitory effects of drugs are determined by the intravesicular accumulation of the probe substrate (usually radiolabeled or fluorescent derivatives of taurocholic acid and other BAs) (Gerloff et al. 1998; van Staden et al. 2012; Yamaguchi et al. 2010). Membrane vesicles from insect cell lines transfected with plasmids to express specific transporters are commercially available and ready to use (Morgan et al. 2010; Yamaguchi et al. 2010). This assay system has been successfully applied to test large series of compounds and to classify/rank order them according to their inhibitory potency (% inhibition, IC50 value) (Morgan et al. 2010; Pedersen et al. 2013). However, their physiological relevance is hampered by excessive simplicity, lack of expression of multiple transporters and their non liver origin. In contrast, canalicular membrane vesicles are more physiologically relevant models, but their widespread use may be limited by technical difficulties to obtain high quality preparations (Horikawa et al. 2003). Therefore, despite membrane-based systems generally being valuable models for the preliminary screening of transport inhibition by drugs, they do not necessary reflect the in vivo situation, and extrapolating results requires taking considerable caution and obtaining confirmation with additional assays that use other experimental models (Pedersen et al. 2013). In fact, it has been reported that these assays can lead to false-positive and false-negative results (Kenna et al. 2018; Kock et al. 2014; Pedersen et al. 2013). Moreover, lack of biotransformation capacity avoids the detection of alterations produced by drug metabolites, and not having multiple transporters and key cellular components makes it practically impossible to obtain valuable information on the mechanisms of cholestasis.

### Cell lines and hepatocyte models

Cell-based models are more physiologically relevant models than membrane vesicles. Diverse epithelial-like cell lines (e.g., human embryonic kidney (HEK293), Madin-Darby canine kidney (MDCK), pig kidney (LLC-PK1) or Chinese hamster ovary (CHO) cell lines) overexpressing a single transporter of interest have been applied to uptake studies (De Bruyn et al. 2011; Kitamura et al. 2008; Mita et al. 2006; Yamashiro et al. 2006). Double transfect cells expressing uptake and efflux transporters are valuable models for transcellular transport studies (Kitamura et al. 2008; Matsushima et al. 2008; Mita et al. 2005). Measuring vectorial transport of substrates across cells that co-express human NTCP and the BSEP are proven useful tools to identify transport inhibition by cholestasis-inducing drugs (Mita et al. 2006). However, as DIC may involve alterations in uptake and/or efflux transporters other than NTCP and the BSEP (Kock et al. 2014; Morgan et al. 2013), the potential inhibition effects of drugs in cell lines double-transfected with other combinations of transporters should also be examined (Matsushima et al. 2008; Yamashiro et al. 2006).

The use of hepatocytes offers several advantages over transfected cell lines, such as their liver origin, the physiological expression of multiple functional transporters and the preservation of normal cellular metabolic capacity, including biotransformation function and regulatory mechanisms. Fresh or cryopreserved hepatocytes in suspension are an easy handling system of uptake assays, but not for efflux studies due to loss of polarization, internalization of efflux transporters during hepatocyte isolation from liver tissue with collagenase and their inability to differentiate between sinusoidal and canalicular effluxes (Badolo et al. 2015; Hoffmaster et al. 2004). Furthermore, suspended hepatocytes remain viable for only a few hours, which is an additional limitation for experimental assessments. These drawbacks can be overcome by culturing hepatocytes in a collagen sandwich configuration that restores tight junctions, bile canaliculi structures, cell polarization and vectorial transport function after a few days in culture (Bi et al. 2006; Hoffmaster et al. 2004; Kostrubsky et al. 2003; LeCluyse et al. 2000; Marion et al. 2007). Sandwich-cultured hepatocytes exhibit many typical liver metabolic functions, including drug-metabolizing enzymes, and have become very popular for evaluating hepatobiliary disposition of endogenous substrates and drugs, estimating transport clearance, identifying regulatory mechanisms, and investigating the role of disruption of BA homeostasis, drug metabolism and alterations of uptake/efflux transport systems in DIC (Ansede et al. 2010; Badolo et al. 2015; Barber et al. 2015; Bi et al. 2006; Donato et al. 2016; Marion et al. 2007, 2012; Ogimura et al. 2011). Several experimental strategies have been applied in sandwich-cultured hepatocytes to distinguish the effects on the transport systems of the basolateral membrane from those of the canalicular membrane. Assay proposals are based on not only the analysis of the accumulation of substrate probes both inside cells and bile canaliculi structures, but also on the estimation of different parameters, such as the biliary excretion index (BEI), bile intracellular correlation (BIC) or in vitro biliary excretion (CL_bile_), to identify cholestatic effects of drugs (Bi et al. 2006; Leslie et al. 2007; Marion et al. 2007; Pedersen et al. 2013). A major shortcoming of this model is the gradual dedifferentiation of hepatocytes over time in culture, which leads to a limited time of use from optimal canalicular networks formation to decline in hepatocyte functions. Furthermore, the restricted availability of high-quality human hepatocytes and variability in functional bile canaliculi structures formation between different batches of cryopreserved hepatocytes significantly affect assay performance and make the interpretation of results difficult.

Human hepatoma cell lines constitute nearly unlimited sources of liver cells and have been proposed as an alternative to human hepatocytes in high-throughput screenings. Of them, HepaRG cells are considered a valuable alternative to human hepatocytes for showing a closer expression of uptake and efflux transport proteins to hepatocytes than in any other hepatoma cell line (Kanebratt and Andersson 2008; Kvist et al. 2018; Susukida et al. 2016). They also mimic many phenotypic features of human hepatocytes, including polarized apical and sinusoidal domains and bile canaliculi networks formation (Bachour-El Azzi et al. 2015; Le Vee et al. 2013). The response of HepaRG to many cholestatic drugs is comparable to that of sandwich-cultured hepatocytes (Sharanek et al. 2015; Susukida et al. 2016; Woolbright et al. 2016). Nonetheless, some discrepancies have been observed that might be attributed to differences in the expression levels of some transport proteins and drug-metabolizing enzymes, and also to the presence of both hepatocyte-like cells (HLCs) and cholangiocyte-like cells in HepaRG cultures (Susukida et al. 2016).

The HLCs generated by differentiation from induced pluripotent stem cells (iPSCs) or by direct linage conversion have emerged as new in vitro models for drug hepatotoxicity screenings and liver disease modeling, including patient-personalized studies of hereditary cholestasis (Hayashi et al. 2021; Li et al. 2022; Lu et al. 2015; Ni et al. 2016). HLCs show many typical features of hepatocytes, such as cell polarization, bile canaliculi-like structures formation, biosynthesis and excretion of BAs and the functional expression of membrane transporters (Lu et al. 2015; Ni et al. 2016; Ulvestad et al. 2013). Compared to primary hepatocytes, HLCs possess an immature phenotype that very much depends on the applied differentiation protocols, and these protocols are often costly and time-consuming (Ni et al. 2016; Ulvestad et al. 2013). Therefore, although HLCs are considered a very promising screening model, their potential usefulness for evaluating the effects of cholestatic drugs has hardly been explored and further studies are needed (Ni et al. 2016).

### Organotypic culture models

Recent technical developments have been directed to optimize cell culture strategies that mimic the spatial organization and multicellular composition of liver tissue. 3D culture models more accurately reflect liver physiology than conventional 2D cultures and stabilize many hepatocyte functions by maintaining a differentiated phenotype over longer culture periods. Human hepatocytes, HepG2 and HepaRG cells maintained as scaffold-free spheroids show polarized organization and the functional expression of canalicular efflux transporters (e.g. MRP2, BSEP) and drug metabolism activities for several weeks (Gunness et al. 2013; Hendriks et al. 2016; Ramaiahgari et al. 2014, 2017). 3D spheroids enable long-term repeated drug exposure studies, show greater sensitivity than 2D cultures in identifying metabolism-dependent toxicity of drugs and recapitulate the mechanisms associated with DICs (Hendriks et al. 2016; Mueller et al. 2014; Ramaiahgari et al. 2017). Similarly, the organoids formed by the self-assembling of human pluripotent stem cells form bile canaliculi networks and show basolateral to apical transporter activity that is altered by cholestasis-inducing drugs (Messina et al. 2022; Ramli et al. 2020; Shinozawa et al. 2021). More complex 3D culture systems, such as micropatterned co-cultures or microfluidic devices, have also been proposed for hepatotoxicity purposes, but their application to DIC studies is still very limited and further studies are required to confirm their usefulness (Zhang et al. 2020a).

Human precision-cut liver slices have also been proposed for cholestasis studies (Vatakuti et al. 2017, 2016). Advantages of liver slices over other in vitro models are the presence of all types of liver cells and the preservation of the native tissue structure with intact cell–cell and cell–matrix interactions. This model may help to reveal the metabolic and signaling mechanisms involved in cholestasis, and the role of non-parenchymal liver cells (e.g., stellate cell activation or Kupffer cell-mediated inflammation) in cholestatic damage progression (Starokozhko et al. 2017; Szalowska et al. 2013; Vatakuti et al. 2017). However, its usefulness for drug screening purposes is hampered by major limitations, such as poor availability of fresh human liver tissue, technical requirements, and short-term maintenance of optimal viability and functional levels.

## Experimental approaches for the prediction of drug-induced cholestasis

Most studies conducted to assess DIC are based on measuring transporter’s functionality or effects on BA accumulation. However, genomics, transcriptomics, proteomics and metabonomics, referred to as the “omics” techniques, offer excellent tools to investigate the fundamental mechanisms that cause DIC. These include hereditary susceptibilities, but also variations in gene expression, protein or metabolite abundance, following drug treatment. In this section, we describe the principal assays used for evaluating DIC.

### Transporter’s function/inhibition assays

Transporter function assays are particularly important when assessing drugs’ cholestatic potential. Liver absorption and biliary excretion of several endogenous and exogenous substances are mediated by transporters and alterations in this transport is one of the major mechanisms implicated in DIC. This means that distinct in vitro assays for measuring biliary excretion and efflux are available.

Fluorescent or radiolabeled BA can be used for calculating the efflux and cumulative uptake of BA (Liu et al. 1999). For instance, [^3^H]taurocholate is a substrate of uptake transporters NTCP and OATPs and efflux transporter BSEP (Bi et al. 2006). The tight connections between bile canaliculi remain intact (closed) when Ca^2+^/Mg^2+^ is present, but become disrupted (opened) when Ca^2+^/Mg^2+^ is absent. Consequently, it is possible to calculate the amount of test chemical in bile canaliculi by measuring the compound’s concentration in both the presence and absence of divalent cations (Bi et al. 2006). By disrupting tight junctions and extending bile canaliculi networks, this calcium-free buffer facilitates the ability to discriminate between cellular and canalicular compartments (Annaert et al. 2001; Liu et al. 1999). The fraction of the accumulated compound that resides in bile canaliculi can be quantified with the BEI (Kemp et al. 2005), whereas the Sinusoidal Excretion Index takes into account the initial loaded taurocholate (Le Vee et al. 2022). Additionally, the apparent uptake rate can also be calculated by the BIC. In fact, the efflux and cumulative absorption of [^3^H]taurocholate in in vitro systems (Liu et al. 1999) or in membrane vesicles (van Staden et al. 2012) using [^14^C]salicylate as a marker for simple diffusion has been widely used. As ABC transporter’s function is ATP-dependent, vehicle controls are carried out in the presence and absence of ATP to guarantee that the substrate is being transported by a particular ATP-dependent pathway. Any radioactivity/fluorescence detected in the controls without ATP is regarded as noise or background (van Staden et al. 2012).

The functional activity of MRP2 can be assessed by analyzing the location of MRP2 model substrate carboxydichlorofluorescein (CDF). Despite having poor fluorescence, CDF diacetate quickly permeates the plasma membrane of hepatocytes and is easily hydrolyzed in the cytoplasm by intracellular esterases to produce highly fluorescent CDF. MRP2 has marked affinity for CDF, which is quickly transferred to bile through the canalicular membrane (Liu et al. 1999).

Cholyl-lysyl-fluorescein (CLF) is a BSEP substrate that has been used as an indicator for BA transport activity (Shinozawa et al. 2021). For instance, CLF transport inhibition induced by different cholestatic drugs has been detected in human liver organoids that derive from iPSC. In fact, the in vitro system has shown high predictivity, and also allows genomic predisposition (CYP2C9*2) for bosentan-induced cholestasis to be detected (Shinozawa et al. 2021).

Finally, the 5-chloromethylfluorescein diacetate (CMFDA) test has been also used as hepatocyte export carrier inhibition assay. CMFDA is a membrane-permeable, non-fluorescent chemical that is converted to fluorescent 5-chloromethylfluorescein by non-specific cytosolic esterases after it enters the cell. 5-Chloromethylfluorescein can be actively exported via membrane transporters like MRP2 and BSEP. Brecklinghaus et al. demonstrated that the use of this assay in cultured human hepatocytes improved the predictivity of the hepatotoxic potential of compounds, including BA export carrier inhibitors (Brecklinghaus et al. 2022).

### BA profile

Total endogenous BA content has been proposed to measure drugs’ cholestatic potential (de Bruijn et al. 2022; Sharanek et al. 2014, 2015, 2017). BA content is usually determined in both cells and culture media by high-performance liquid chromatography/mass spectrometry, and the ratio is calculated. In addition to total BA production and profiles, BA conjugation has also been analyzed under different experimental conditions (de Bruijn et al. 2022; Sharanek et al. 2014, 2015, 2017). In fact in studies that have employed differentiated HepaRG cells, the most powerful cholestatic drugs (i.e. cyclosporin A, troglitazone, chlorpromazine) induce the preferential intracellular accumulation of unconjugated forms of CDCA, LCA, and DCA in the presence of physiological serum BA concentrations (de Bruijn et al. 2022; Sharanek et al. 2014, 2015, 2017). This renders them potential biomarkers of cholestatic drugs (Sharanek et al. 2014). Therefore, it is essential to add BA to the medium to detect BA accumulation in vitro. In fact, under these conditions, cholestatic drugs can induce cellular BA accumulation without significant cellular damage, which is consistent with the extended survival of hepatocytes in cholestatic livers regardless of high BA levels (Burban et al. 2019).

When comparing different in vitro models, the de novo synthesis rate of BA in sandwiched-cultured hepatocytes was the highest of three studied hepatic in vitro models (sandwiched-hepatocytes, HepaRG, intrahepatic cholangiocyte organoids), whereas intrahepatic cholangiocyte organoids showed very low BA production (de Bruijn et al. 2022). Other authors have proposed a biokinetic model to measure intracellular BA (Notenboom et al. 2018). Based on human in vitro data, they created a mechanistic biokinetic model that mimics intrahepatic BA concentrations in both the presence and absence of cholestatic drugs. Those authors measured and included the kinetic data of two selected BAs (CDCA, DCA), and their glycine and taurine conjugates, and then provided a method that is able to indicate if drugs induce cholestasis by inhibiting the BSEP (Notenboom et al. 2018).

### Cholestatic index

The Cholestatic Index (CIx) is a measure of the degree of interference of additional BA in the presence of the suspected drug to assess the cholestatic risk. The CIx is computed by assessing the combined effects of a BA mixture on the cytotoxicity elicited by a test chemical. For this purpose, the EC50-value from co-exposure to a chemical and a selected BA mixture divided by the EC50-value from exposure to the compound alone, is known as the CIx (Hendriks et al. 2016). To this end, different tests have been proposed, such as effects on viability or urea production in the absence or presence of BA (Deferm et al. 2019b; Hendriks et al. 2016). For the BA mixture, several combinations of the commonest BA and concentrations have been proposed. They range from physiological conditions (Hendriks et al. 2016) to concentrations up to 30-, 60-, or even 100-fold, the maximum plasmatic concentration values (Rose et al. 2022; Sharanek et al. 2017; Vilas-Boas et al. 2021) to see differential effects when treating cell models with suspected cholestatic drugs in the presence or absence of BA.

### Transcriptomics

Transcriptomics profiling is widely utilized to detect DIC (Gijbels et al. 2021; Parmentier et al. 2017; Vatakuti et al. 2017, 2016). Understanding alterations in the transcriptome upon exposure to a drug with a cholestatic potential can offer major mechanistic comprehension, which can be key for the safety assessment of novel chemical entities (Kralj et al. 2021). An established transcriptome signature of cholestatic DILI, which includes changes in the genes related to inflammation (i.e. c-jun*,* interleukin 1 receptor type 1*,* serpin E1, superoxide dismutase 2), oxidative stress (i.e. NRF2 pathway), ER stress (transcription factor 4/6, DNA damage inducible transcript 3 and heat shock protein family A5), various forms of cell death and the adaptive response in cholestasis has been described by Gijbels et al. (2020a), and has been applied in several studies (Gijbels et al. 2021, 2020b).

Other authors have described how BSEP gene expression is impaired by different cholestatic-associated drugs, such as troglitazone or bosentan, in an FXR-dependent manner (Garzel et al. 2014; Vatakuti et al. 2017). Actually, based on how test compounds affected the BSEP expression in cultured human hepatocytes, the authors categorized substances into three groups: potent, moderate and negligible repressors (Garzel et al. 2014). The gene expression profile induced by cholestatic drugs has also been comparatively analyzed in several in vitro models (Gijbels et al. 2021; Van den Hof et al. 2017) and compared to data from patients with cholestasis, although this was not caused by drugs (Van den Hof et al. 2017).

### Metabonomics

Metabolites’ profile has been also pointed out for the identification of cholestatic features. For instance, apart from the down-regulation of different BAs as an adaptive mechanism, a decrease in phospholipid precursors has also been described in DIC (Cuykx et al. 2019). A metabolomics study of the cell supernatants of HepaRG cells treated with bosentan has identified significant changes in 3-methyl-2-oxovalerate, carnitine, acetate, acetoacetate and lactate, as well as a decrease in the absorption of amino acids (namely leucine and isoleucine). This scenario reflects mitochondrial dysfunction induced by cholestatic drugs (Rodrigues et al. 2018). Van den Hof et al. (2015) have described an integrated approach that combined the study of significant changes in mRNAs and metabolites separately and integrated after treating HepG2 cells with Cyclosporin A (Van den Hof et al. 2015). Most of the pathways that were only discovered through the integrated analysis were associated with cellular mechanisms, which were also discovered through separate analyses. These mechanisms included the cell cycle, cholesterol and lipid metabolism, and pathways that were significantly enriched in detoxification, transport and ER protein processing. Only one or two of the pathways involved in glucose and biliary homeostasis were identified by separate studies, but the integrated analysis revealed many pathways engaged in these processes (Van den Hof et al. 2015).

### Proteomics

Proteomics has also been used to corroborate the transcriptomics findings of bosentan-induced cholestasis in HepaRG cells (Rodrigues et al. 2018). According to a functional pathway analysis of the identified proteins, the HepaRG cells treated with bosentan exhibited an enrichment of toxicological classes “cholestasis” and “intrahepatic cholestasis”, which points out the usefulness of this technique for identifying the cholestatic cellular response (Rodrigues et al. 2018).

### miRNoma

microRNAs (miRNAs) are a class of small non-coding RNAs that regulate gene expression post-transcriptionally. Changes in miRNA expression have also been linked with DIC (Wolters et al. 2016). Those authors performed a multi-omics experiment after cyclosporin treatment for 3 and 5 days, which included whole genome methylation, gene expression and an miRNA expression analysis. After cyclosporin treatment, miR-186-5p, miR-4659a-3p, miR-630, miR-5703, miR-5787 and miR-6129 showed an increase, while miR-let-7e-5p, miR-30a-3p and miR-324-5p displayed a reduction (Wolters et al. 2016). Interestingly, six persistent differentially expressed miRNAs showed an agreement with 22 targeted differentially expressed genes that remained expressed even after stopping drug treatment. Of these, it was discovered for the first time that liver genes, such as *HERPUD1, NUCB2* and *LEAP2* and related miRNAs (hsa-let-7e-5p and hsa-mir-5787), were consistently up- or down-regulated (Wolters et al. 2016).

### High-throughput assays

In order to increase the throughput of assays to measure drugs’ cholestatic potential, an alternative technique that visualizes biliary CLF excretion by a high-content screening imaging algorithm has also been proposed (Barber et al. 2015). Those authors used sandwich-cultured rat hepatocytes and tested 41 compounds. They demonstrated that 29 of them inhibited biliary CLF efflux and a wide variety of inhibitory potencies was seen (Barber et al. 2015). One of this method’s major advantages was that it does not require the use of radiolabelled substrates, and it utilizes direct live cell measurements of biliary excretion in vitro. As cell viability can be evaluated in the same samples employed for imaging, this method can directly demonstrate that the impact of the test compounds on canalicular efflux are not attributable to cytotoxicity.

### Other assays

Seeing that different mechanisms have been implicated in DIC, other assays like measuring ROS production have been suggested as potential assays for determining drug’s cholestatic potential. For instance, Sharanek et al. measured ROS production and oxidative stress and ER stress-related genes to unravel the mechanisms underlying DIC (Sharanek et al. 2014). Other authors have analyzed changes in the mitochondrial membrane potential, ROS production or caspase-3 activity to gain a mechanistic understanding of DIC (Ni et al. 2016).

BSEP inhibitors have been modeled using a variety of computational techniques, such as the quantitative structure–activity relationship analysis, to associate molecular descriptors with BSEP inhibition (Kenna et al. 2018). However, the identification of common structural features is challenging because of the structural diversity of the drugs that cause BSEP inhibition (Kenna et al. 2018).

Recently, a combination of omics data and in silico approaches has attempted to provide a better comprehension of the molecular mechanisms of DIC. For instance, Jiang et al. developed a bioinformatics tool consisting of applying machine-learning algorithms to the Open TP-GATEs database to identify a transcriptomic signature related to DIC (Jiang et al. 2023). By applying this approach to human hepatocytes exposed to cholestatic and non-cholestatic drugs, a set of 13 genes mechanistically related to membrane transport function, BA metabolism, inflammation, ROS production or ER stress was linked with DIC.

## Future perspectives

The early and precise detection of drugs’ cholestatic potential and chemicals is expected to be made possible by the full integration of new knowledge and technologies in years to come. DIC is a multifactorial phenomenon with various drug- and patient-related factors that may influence the clinical phenotype (pure or mixed cholestasis) and outcome (acute or chronic cholestasis, final recovery or fatality). Therefore, a better comprehension of the DIC pathophysiology depends on a global understanding of the molecular mechanisms and individual risk factors that cause this disorder, which would help to develop safer drugs. Moreover, it should be considered that other organs may be considerably affected by cholestasis. A serious side effect of cholestasis and an unmet therapeutic need is cholemic nephropathy, an acute kidney injury that can be caused by the accumulation of BA in the systemic circulation. In fact, it has been recently shown that BAs are specifically absorbed by the kidney's proximal tubular epithelial cells through ASBT and that blocking renal ASBT specifically protects tubular epithelial cells (Ghallab et al. 2024). Thus, other side effects of cholestatic drugs should be also considered and analyzed when evaluating the toxic potential of drugs under development.

Although damage to hepatobiliary transporters is one of the major mechanisms of DIC, and most methods have relied on assessing a transporter’s functionality, mixtures of different endpoints should be used preferably to single assessments. Moreover, it is preferable for cell-based methods to be supplemented with computational techniques that use structural or physico-chemical parameters to predict drugs’ cholestatic potential. In addition, prescription regimes and pharmacokinetic properties of drugs, particularly drug bioavailability and their potential intrahepatic accumulation, should be considered and, thus, physiologically based kinetic models could be a valuable contribution. Overall, by combining in vitro and in silico approaches will undoubtedly increase the predictivity of DIC in early drug discovery phases.

Given the vast variability of DIC events, in vitro methods capable of reproducing such interindividual differences are promising for the possible evaluation of individual susceptibility. As primary liver tissue harvesting is inefficient and challenging, iPSC-derived models will be a useful tool for precision research. iPSC-derived models enable the way genetic variation affects the systems involved in drug metabolism and transport to be assessed, and how this relates to adverse effects, as well as the potential contribution of gene polymorphic variants of BA transporters.

## Abbreviations

ABC: ATP-binding cassette
ASBT: Apical sodium-dependent bile salt transporter
BAs: Bile acids
BEI: Biliary excretion index
BIC: Bile intracellular correlation
BSEP: Bile Salt export pump
CDCA: Chenodeoxycholic acid
CDF: Carboxydichlorofluorescein
CIx: Cholestatic index
CLF: Cholyl-lysyl-fluorescein
CMFDA 5: Chloromethylfluorescein diacetate
CYP7A1: Cholesterol 7α-hydroxylase
DCA: Deoxycholic acid
DIC: Drug-induced cholestasis
DILI: Drug-induced liver injury
ER: Endoplasmic reticulum
FXR: Farnesoid-X receptor
HLCs: Hepatocyte-like cells
iPSC: Induced pluripotent stem cells
LCA: Lithocholic acid
MDR: Multidrug resistance protein
miRNAs: MicroRNAs
MRP: Multidrug resistance-associated protein
NTCP: Na^+^taurocholate cotransporting polypeptide
OATP: Organic anion transporting polypeptide
OST: Organic solute transporter
ROS: Reactive oxygen species
SHP: Small heterodimer partner
